# The Free Energy Landscape of Dimerization of a Membrane Protein, NanC

**DOI:** 10.1371/journal.pcbi.1003417

**Published:** 2014-01-09

**Authors:** Thomas A. Dunton, Joseph E. Goose, David J. Gavaghan, Mark S. P. Sansom, James M. Osborne

**Affiliations:** 1 Department of Computer Science, University of Oxford, Oxford, United Kingdom; 2 Department of Biochemistry, University of Oxford, Oxford, United Kingdom; Max Planck Institute for Biophysical Chemistry, Germany

## Abstract

Membrane proteins are frequently present in crowded environments, which favour lateral association and, on occasions, two-dimensional crystallization. To better understand the non-specific lateral association of a membrane protein we have characterized the free energy landscape for the dimerization of a bacterial outer membrane protein, NanC, in a phospholipid bilayer membrane. NanC is a member of the KdgM-family of bacterial outer membrane proteins and is responsible for sialic acid transport in *E. coli*. Umbrella sampling and coarse-grained molecular dynamics were employed to calculate the potentials of mean force (PMF) for a variety of restrained relative orientations of two NanC proteins as the separation of their centres of mass was varied. We found the free energy of dimerization for NanC to be in the range of 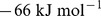 to 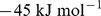. Differences in the depths of the PMFs for the various orientations are related to the shape of the proteins. This was quantified by calculating the lipid-inaccessible buried surface area of the proteins in the region around the minimum of each PMF. The depth of the potential well of the PMF was shown to depend approximately linearly on the buried surface area. We were able to resolve local minima in the restrained PMFs that would not be revealed using conventional umbrella sampling. In particular, these features reflected the local organization of the intervening lipids between the two interacting proteins. Through a comparison with the distribution of lipids around a single freely-diffusing NanC, we were able to predict the location of these restrained local minima for the orientational configuration in which they were most pronounced. Our ability to make this prediction highlights the important role that lipid organization plays in the association of two NanCs in a bilayer.

## Author Summary

Cells are surrounded by selectively-permeable bilayer membranes, enabling the cell to control its internal environment. Embedded within these membranes are a variety of membrane proteins, many of which facilitate this environmental control and are integral to numerous metabolic processes. Their location within the membrane and their mutual association are controlled by many factors. We use molecular dynamics simulations to investigate the free energy of association for a pair of relatively simple membrane proteins. By doing so, we are able to characterize the effect that the geometrical properties of the protein have on their mutual association in a bilayer environment, showing that there is a correlation between the buried surface area of two proteins when in contact and the strength of their interaction. We also observe the effect of protein-lipid-protein interactions in this free energy characterization. Such interactions are related to the preferential distribution of lipids around proteins in the membrane.

## Introduction

Cellular membranes not only separate the contents of a cell from its surroundings, they also play a key role in cell regulation and metabolism. Accounting for approximately a quarter of the coding regions of an organism's genome [1], membrane proteins control the transport of solutes between a cell and its surroundings, facilitate cellular movement, and regulate many aspects of cellular behaviour.

Gram-negative bacteria are surrounded by two membranes separated by a periplasmic layer. The outer membrane lipid bilayer is composed of phospholipids in the inner (i.e. periplasmic) leaflet, and of lipopolysaccharides in the outer leaflet. Within this membrane are many species of outer membrane proteins (OMPs), a class of integral membrane proteins whose secondary structures are almost exclusively 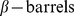 [2]. Many of these 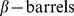 are porins (OmpC, OmpF, LamB, NanC, for example), through which small (approximately 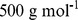) molecules can diffuse across the membrane. Porins provide a route for many antibiotics into bacterial cells and are potential vaccine targets [3].

Both *in vivo* and *in vitro*, membrane proteins are often present in a crowded environment. Thus, cell membranes generally have a high membrane area fraction (approximately 25% or greater) occupied by proteins [4]. A similar degree of crowding may be found in membranes studied *in vitro* [5], [6]. Such crowding may result in the clustering of proteins [7]. Whilst the majority of discussion as to the nature of membrane protein cluster formation has focussed on lipid rafts [8], it should be noted that lateral interactions of crowded membrane proteins are a more general property [9]–[12]. *In vitro*, control of lateral association of a single membrane species in a highly crowded system may be used to induce two-dimensional crystallization [13].

**Figure 1:**
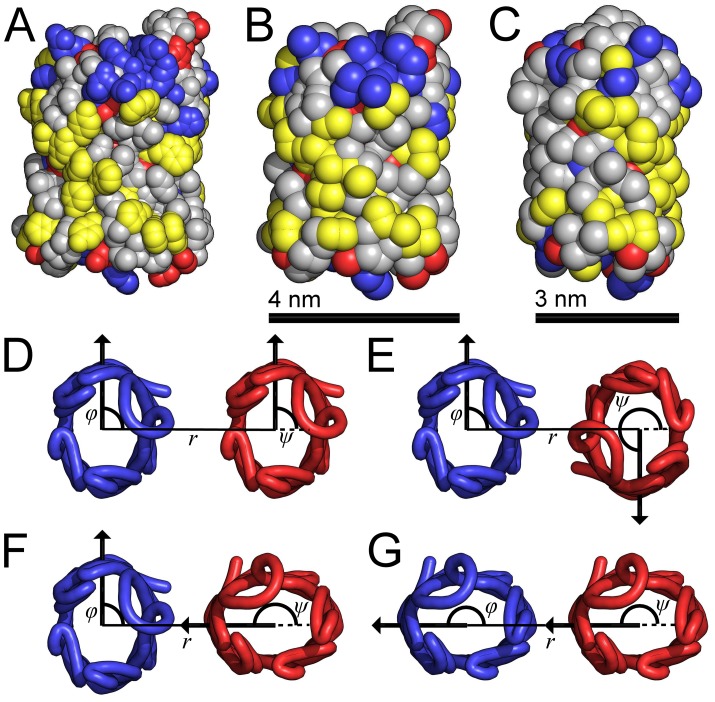
Atomistic and coarse-grained representations of NanC along with illustrations of the orientational combinations used in the PMF calculations. (*A*) The atomistic structure of NanC is shown in the plane of the bilayer, which is perpendicular to the pore axis. (*B*–*C*) The coarse-grained NanC is shown in both the wide (*B*) and narrow (*C*) orientations, which are related by a 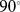 rotation about the pore axis and with *B* being the equivalent orientation to *A*. The atoms/particles are represented by spheres with radii equal to their van der Waals radii. The atoms/particles from acidic residues shown in red, from basic residues in blue, from aromatic residues in yellow and from neutral residues in grey. (*D*–*G*) Four combinations of protein orientations as viewed from the extracellular side of the membrane. The 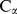 traces of the NanC proteins illustrate their elliptical cross-sections. For each protein, the angle of orientation is measured between the line, which goes from the centre of mass of the blue protein through the centre of mass of the red protein, and the arrow, which goes from the protein's centre of mass through the 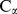 of its isoleucine at residue 209. The orientational angle for each protein trace coloured blue is labelled 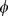 and for each protein coloured red is labelled 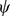. The separation between the proteins' centres of mass is given by 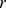.

Interactions within a crowded environment may lead to dynamic lateral interactions of membrane proteins, for example, those seen in recent time-resolved AFM studies of OmpF-containing membranes [6]. In studying such interactions, one wishes to distinguish between specific oligomerization of membrane proteins (for example dimerization of transmembrane 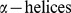 in glycophorin [14] and of 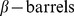 in OMPLA [15]) and less specific interactions. Specific protein interactions are those in which the distributions of orientations of the oligomerized proteins are grouped almost exclusively into very few states (often only a single state). Less specific (or non-specific) interactions are those determined by other effects, such as (local) crowding, rather than purely due the specific interactions between residues on each of the proteins. In less specific oligomerization there may be some orientational dependence, but a more broad distribution of orientations is generally found among the oligomers. Benjamini and Smit found that it was important to determine the effect that non-specific interactions had on the crossing angle for pairs of 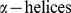 before investigating the role of any specific interactions between the helices [16]. It is therefore of interest to explore the energy landscape of lateral interactions of a relative ‘featureless’ OMP. NanC (Figure 1A) provides a good example of such a protein, as it is both structurally [17] and functionally [18] monomeric, whilst forming two dimensional crystals in DMPC lipid bilayers [19]. NanC is member of the KdgM-family of bacterial outer membrane proteins, responsible for sialic acid transport in *E. coli*.

Experimental determination of the free energy of membrane protein dimerization *in vitro* has been used to characterize their properties in a membrane or membrane-like environment [20]–[25]. Characterization of the free energy landscape for membrane proteins gives us an insight into how the proteins will move and interact within the membrane and allows us to make predictions about their behaviour. There are many published examples of experimentally determined dimerization energies for 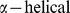 membrane proteins and peptides [20]–[22], [24], [25], but relatively few for 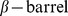 proteins: one important example being the dimerization free energy of the phospholipase OMPLA, which was found to be in the region 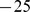 to 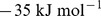 [23].

Computer simulations provide a complement to both *in vitro* and *in vivo* experiments [26], enabling us to probe the microscopic interaction underlying membrane protein association. Molecular dynamics (MD) simulations have been used to explore a range of membrane proteins [26], in addition to related approaches such as Monte Carlo [27] and Brownian dynamics [28] simulations. In particular, simulations using a coarse-grained approximation [29] have been used to study dimerization of 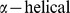 transmembrane domains [30] and of rhodopsin [31]. In the latter case the simulations were also used to characterize large-scale organization of rhodopsin dimers into rows-of-dimers, as seen experimentally in disk membranes.

Many computational studies that explore free energy landscapes use the potential of mean force (PMF) [32] as a convenient description because it enables us to characterize a given reaction or transition as a function of a specific reaction coordinate (or set of coordinates). Not only does this enable us to characterize the free energy landscape as a function of the reaction coordinate (or coordinates), it also provides an opportunity subsequently to parameterize reduced models of complex systems using different simulation paradigms [33].

Calculations of PMFs for the association of membrane proteins have largely focussed on 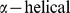 proteins. Thus, dimerization free energy landscapes for transmembrane 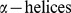 have been calculated using MD with umbrella sampling [30], [34] and with adaptive biasing force methods [35]; and also using Monte Carlo [27], and dissipative particle dynamics [36]. These have yielded free energies of dimerization in the region of 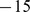 to 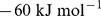. To date there has only been one computational study to calculate the association free energy of a 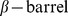 membrane protein: the free energy of association for two OmpF trimers was estimated to be in the region of 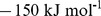 [6].

It has long been suggested that lipids play an important role in the interaction between proteins in a membrane [37], [38]. For example, simulation studies have shown that hydrophobic mismatch may modulate the aggregation of proteins in the membrane [39], [40]. It is therefore important that we capture the effects that lipids have on free energy landscapes if we are to understand membrane protein association in different bilayer environments.

In this paper we develop and apply a method for calculating the free energy of association for rotationally restrained proteins in a lipid bilayer. This allows us to resolve detailed structure in the (one-dimensional) PMFs, which reflect protein-lipid-protein interactions. We apply this method to characterize the association free energy of a coarse-grained model of NanC.

## Results

In order to characterize the proteins' free energy of association, we employed MD simulations of a coarse-grained model of NanC in a POPE bilayer to calculate the PMF [29], [41]. Two orthogonal orientations of our coarse-grained NanC are shown in Figures 1B and C. It can be seen from these two views of the coarse-grained protein that, perpendicular to the pore axis, the protein is approximately elliptical in cross section, as it is wider in one direction (Figure 1B) than it is in the other orthogonal direction (Figure 1C). We calculated the PMF for four different orientational configurations of two NanC proteins, as shown in Figures 1D–G. These four configurations were chosen as examples of extremes of the possible contact regimes between the two proteins on association. In each of the configurations the proteins have either a wide (e.g. Figure 1B) or narrow face (e.g. Figure 1C) facing the other protein. These orientational configurations are categorized as: two configurations corresponding to maximal protein contact, where wide faces of both proteins face the other protein (shown in Figures 1D and E); one intermediate configuration, in which a narrow face of one protein faces a wide face of the other protein (shown in Figure 1F); and one configuration corresponding to minimal contact, where narrow faces of both proteins face the other protein (shown in Figure 1G). From these combinations of protein orientations we were able to investigate the differences between the PMFs for the various protein contact regimes.

### Potentials of mean force

**Figure 2:**
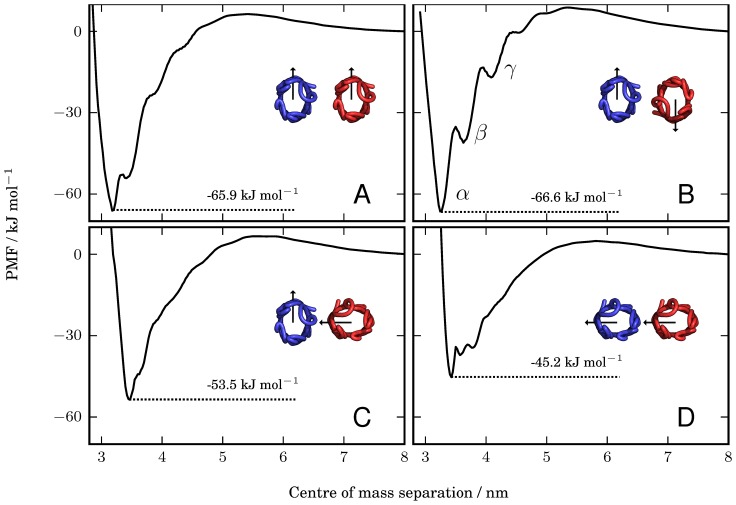
Potentials of mean force for four orientational configurations of the NanC proteins. The four combinations are for angles of: 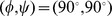; 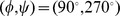; 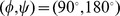; and 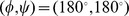 (in *A*, *B*, *C*, and *D*, respectively). The PMFs have depths of 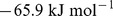 (*A*); 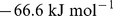 (*B*); 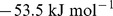 (*C*); and 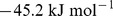 (*D*). In *B*, the minimum, labelled 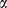, and the two local minima, labelled 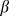 and 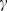, correspond to inter-protein separations at which different numbers of lipids can optimally occupy the intervening region between the two proteins, as explained in the main text and illustrated in Figure 3.

The PMFs calculated for each of the four rotational combinations are shown in Figure 2. The PMFs were set to zero at an inter-protein separation of 8 nm, where the potentials have become approximately constant. The sampling methods, biasing potentials, rotational restraints and simulation details are given in the Methods section.

We categorized the PMFs in Figure 2 by the depths of their potential well, which resulted in three categories of well depth. The first category contains the PMFs in Figures 2A and B, which both have depths of approximately 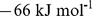 occurring at inter-protein separations of approximately 3.2 nm. This first category corresponds to the orientational configurations of maximal contact, 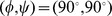 and 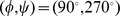, where wide faces of both proteins are brought into contact (shown in Figures 1D and E, respectively). It is interesting to note that the depths of the PMFs for these two parallel and anti-parallel orientational configurations are approximately the same. They are also similar in depth to the orientationally-unrestrained PMF calculated for this coarse-grained NanC system (shown in Figure S2), which has a depth of 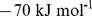. This is much greater than the 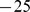 to 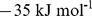 calculated for the dimerization of OMPLA [23], the only experimental free energy for dimerization of an OMP, but as that was for a protein exhibiting specific oligomerization measured in detergent micelles, we would not expect a good agreement. The next category contains the PMF in Figure 2C, with a potential well depth of approximately 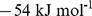 occurring at a separation of approximately 3.5 nm. This corresponds to the intermediate orientational configuration in Figure 1F, where a wide face of one protein is brought into contact with a narrow face of the other. The decrease in the depth of the PMF indicates that the configurations with two wide faces in contact are more stable than this intermediate contact configuration, where 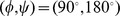. The third category contains the PMF shown in Figure 2D, which is the shallowest of the four PMFs with a potential well depth of approximately 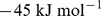, occurring at an inter-protein separation of 3.5 nm. This PMF corresponds to the orientational configuration with minimal protein contact, where narrow faces of both proteins are brought into contact (shown in Figure 1G). This configuration is the least stable of the four configurations considered here. The correlation between the depth of the PMFs and the orientational configuration of the proteins suggests that the strength of the interaction may correlate with the overall extent of the resultant protein-protein interface.

### Restrained metastable states are observed at various inter-protein separations

As well as the restrained global minima (the global minima for the specific restrained orientations) of the potential wells in the PMFs of Figure 2, there are also multiple local minima, which occur at a variety of centre of mass separations. For example, the PMF in Figure 2B has a restrained global minimum (labelled 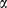) and two higher-energy local minima (labelled 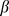 and 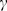), which we refer to as restrained metastable states. By fitting quadratic curves to the minima in Figure 2B we calculated their locations as 3.26 nm, 3.62 nm and 4.07 nm for 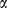, 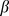 and 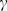, respectively.

**Figure 3:**
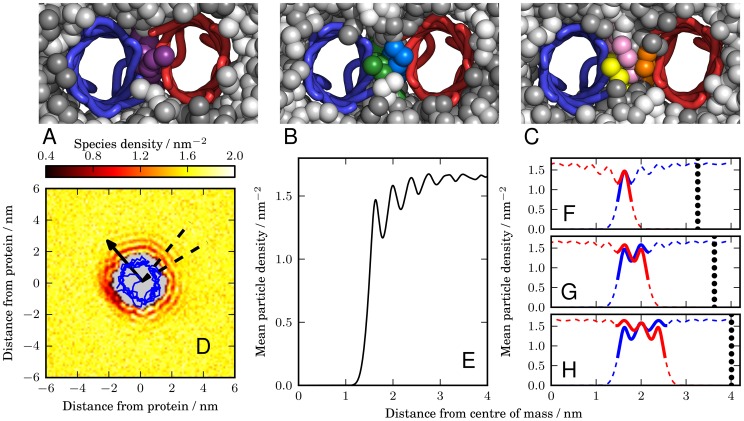
Restrained metastable states in the PMFs can be explained in terms of the protein-lipid-protein interactions that result from the lipid ordering between the two proteins. (*A*–*C*) Snapshots taken from umbrella sampling windows for the orientational configuration 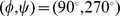 at inter-protein separations of 3.3 nm, 3.6 nm, and 4.1 nm, respectively. These snapshots correspond to the local minima in Figure 2B, which are labelled 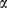, 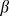 and 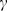. The membrane is viewed from the periplasmic face, with the water and ions removed. The proteins are represented by traces through the 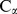 particles of each residue, with one protein coloured blue and the other red. The lipid molecules are represented by spheres and are coloured by the lipid molecule, so that individual lipids can be identified. We see in the snapshot of state 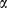 (*A*) that there is only one lipid between the two proteins, which is coloured purple. In the snapshot of state 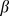 (*B*) there is room for two lipid molecules, coloured green and blue, to fit between the two proteins. In the snapshot of state 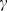 (*C*) there is room for three lipids to fit between the two proteins, coloured in yellow, pink and orange. (*D*) Species density plot for the third coarse-grained particle in one of the tails for the lipids in the upper leaflet. The density is measured relative to the position of a freely diffusing NanC protein. The blue line is a projection of the 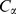 particles onto the plane of the membrane. The arrow is the same arrow used throughout the text to show the protein's orientation and is drawn from the centre of mass through the 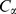 of the isoleucine at residue 209. The dashed lines mark the angular region over which the mean lipid density (*E*) is measured and corresponds to the direction of the other protein in the orientational configuration 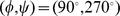. The mean is taken over both leaflets and all coarse-grained lipid particles. (*F*–*H*) The distribution is overlaid with a reversed version and aligned such that either one (*F*), two (*G*), or three (*H*) peaks occur in the region between the two proteins. The dashed line sections correspond to regions that are occupied by the proteins. The thick lines represent the overlaid lipid distributions around both proteins that correspond to the prediction of the lipid packed region between the two proteins. The vertical dotted lines indicate the edge of the reversed density plot. These edges correspond to the predicted position of the second protein and are located at 3.24 nm (*F*), 3.63 nm (*G*), and 4.02 nm (*H*).

The nature of the restrained global (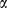) and local (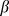 and 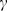) minima is illustrated by the simulation snapshots shown in Figures 3A–C. These snapshots were taken from the simulation windows used to calculate the PMF in Figure 2B for an orientational configuration of 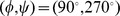. The snapshot shown in Figure 3A is from the umbrella sampling window in which the proteins were restrained with a separation of 3.3 nm, which is closest to the minimum at 3.26 nm in Figure 2B, labeled 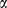. We see that there is one lipid molecule between the two proteins at this restrained global minimum. It should be noted that this is the only lipid in between the two proteins; there is no equivalent lipid on the extracellular side of the membrane (the view from the other side of the membrane is shown in Supporting Information Figure S1), so the restrained global minimum configuration for this orientation has space for one lipid on the periplasmic side of the membrane. A snapshot from the umbrella sampling window with the proteins restrained with a separation of 3.6 nm is shown in Figure 3B, which is the window closest to the minimum at 3.62 nm, labelled 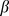 in Figure 2B. We can see that there are two lipid molecules between the two proteins in this snapshot. The snapshot in Figure 3C is taken from the umbrella sampling window in which the proteins are restrained with a separation of 4.1 nm, which is the window closest to the minimum at 4.07 nm, labelled 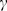 in Figure 2B, in which we see that three lipid molecules can occupy the space between the two proteins. These observations suggest that the existence of these restrained metastable states is a result of protein-lipid-protein interactions in this orientationally-restrained system.

To investigate the suggestion that these restrained metastable states were the result of the lipid ordering between the proteins, we calculated the lipid distribution around a freely diffusing NanC in a POPE bilayer. The distribution for a specific coarse-grained particle in the tail of all of the lipid molecules is shown in Figure 3D, where distinct annuli are visible, indicating regions of preferred occupation. We calculated the lipid distribution in a direction that corresponds to the direction of the other protein for the orientational configuration 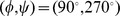, indicated by the region between the dashed lines in Figure 3D. The average lipid distribution across both leaflets and all coarse-grained lipid particles in this direction is shown in Figure 3E, where again we can see there are preferred distances from the protein at which the lipids are observed. Further details of the averaging calculation are given in the Methods section.

We can use this directional lipid distribution to predict the separations at which the region between two proteins would be optimally packed by the lipid molecules. The alignment process is illustrated in Figures 3F–G and explained in the Methods section. For the minimum labelled 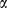 in the PMF in Figure 2B, which occurs at a separation of 3.26 nm, we predict an optimal separation of 3.24 nm with one intervening lipid. For the first restrained metastable state labelled 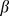 in Figure 2B, which occurs at a separation of 3.62 nm, we predict a separation of 3.63 nm with two intervening lipids. For the second restrained metastable state labelled 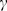 in Figure 2B, which occurs at a separation of 4.07 nm, we predict a separation of 4.02 nm with three intervening lipids. Our predictions for the locations of the restrained metastable states are in close agreement with their location in the PMF. This supports our suggestion that the restrained metastable states observed in the PMFs are due to the protein-lipid-protein effects caused by the distribution of lipids between the two NanC proteins. For the other orientational configurations, the proteins have different faces facing the other protein and will therefore have a different optimal lipid distribution for each face. This may be one reason why the restrained local minima are better defined for 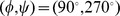 and occur at regular intervals.

Such features are not usually observed in PMFs calculated with proteins that are free to rotate (for example, see Figure S2 for an orientationally-unrestrained PMF calculated for this coarse-grained NanC system). In the orientationally-unrestrained case the proteins would be able to rotate to alter the distance between their surfaces, provided they are not perfectly rotationally symmetric, so that the intervening region could be optimally packed with lipids without leaving any voids. However, for a system with rotationally restrained proteins, there is an optimal separation at which multiple lipid molecules can occupy the intervening space between the proteins.

Also observed in each of the PMFs is an energetic barrier, which occurs at an inter-protein separation of approximately 5.5 nm. Extending the arguments made above about the interaction of the two proteins individual lipid distributions, we can see that at distances greater than 2.5 nm in Figure 3E the fluctuations in lipid distribution have decayed to small oscillations around some constant average value, which indicates that these lipids are not as strongly influenced by the protein. From this argument we can think of this barrier as the point at which the lipids whose positions are strongly dependent on each protein begin to interact with one another, that is, there are lipids between the proteins that are affected by both of the proteins. We can think of this as the separation at which the annuli of lipids around each protein overlap with each other.

### Analysing the lipid-inaccessible buried surface area

We wished to formally characterize the dependence of the PMF depth on the orientation of the proteins, which we suggested was related to the extent of protein contact. To do this we calculated the solvent accessible surface area (SASA) of the two proteins as a function of the separation of their centres of mass. For proteins with an approximately elliptical cross-section, we would expect the orientations with greater contact between the proteins to have a larger buried surface area. However, given the significance of the lipid effects that we identified above, it is important to corroborate this. Any features of the combined surfaces of the two proteins that would allow room for a lipid could have a large effect on the free energy.

The SASA is calculated using a spherical probe whose size determines the level of detail in the surface calculated for a specific set of atoms/particles. We used a probe with a radius of 0.47 nm, which is twice the radius of the coarse grained particles (0.235 nm) and should be a reasonable measure for the size of a lipid. We chose this size probe because it is the lipids that are the ‘solvent’ of interest when we bring two proteins together in a membrane. Further details are given in the Methods section.

**Figure 4:**
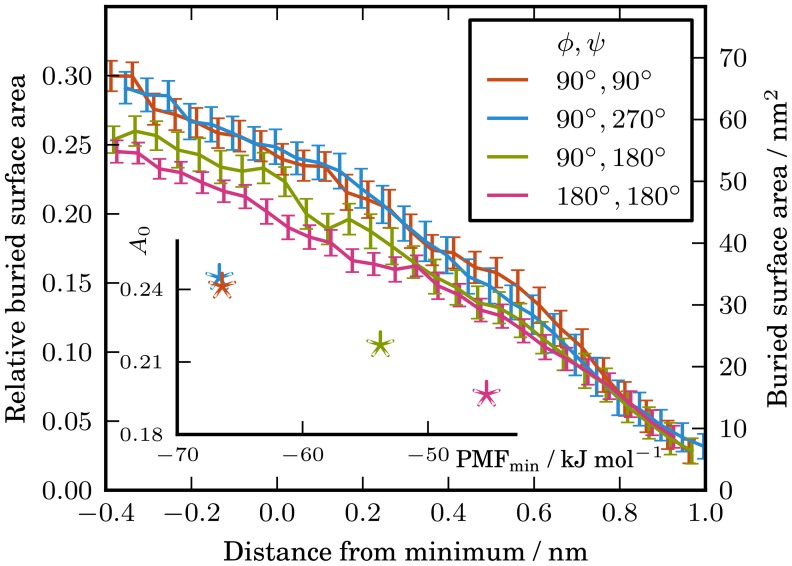
Buried surface area of the protein complex as a function of distance from the minimum of the PMF. The error bars correspond to 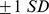. The inset figure shows the relative buried surface areas at the minimum of the respective PMFs as a function of the restrained global minimum PMF depth. The buried surface areas at the minimum of the PMFs were obtained from a straight line fit of the region 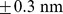 from the minimum of the PMF.

For each of the four orientational configurations, Figure 4 shows the buried surface area as a function of distance from the minimum of their respective PMFs. We chose this measure since we wanted to remove the effect that the difference in protein radius has on the location of the minimum. In Figure 4 we see that there is a stratification of the buried surface areas in the region around the minima of the PMFs. As with the PMF depths in Figure 2, the buried surface areas can be divided into three categories. The buried surface area is largest for the orientations 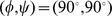 and 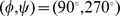, where the two wide protein faces are brought together. The next largest buried surface area around the minimum of the PMF is for the orientation 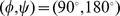, where one narrow face is brought into contact with one wide face. The smallest buried surface area around the minimum of the PMF is for the orientation 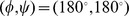, where two narrow faces are brought into contact. The correlation between the depth of the PMF and the buried surface area can be seen in the inset plot in Figure 4, in which these two quantities are plotted. We see that there is a negative correlation between the two quantities. For a protein orientation with a larger buried surface area, the minimum of the PMF is deeper.

## Discussion

We have characterized the free energy landscape of a pair of NanC proteins in a phospholipid bilayer. An interesting feature of these restrained free energy calculations is that certain restrained metastable states, which would usually not be seen, are now resolved. These local minima are associated with the ability of lipids to occupy the space between the two proteins at a given separation. Niemelä et al. [42] found that close to proteins, the lipids in a bilayer have reduced mobility, diffusing with the protein, and it is interactions involving these surrounding lipids upon protein association that we are observing here. Proteins' interactions with lipids have been shown to modulate local lipid formation [43], further demonstrating the important role of interactions involving both proteins and lipids in determining structures observed in the bilayer. PMFs for the dimerization of TM helices [27], [30] and for other more complex proteins, including rhodopsin [44] and OmpF [6] have revealed similar features, suggesting that a role for lipids in the energetics of their interactions may be a general feature of membrane proteins. These features may not affect the kinetics of association, as we do not know if they present metastable barriers to association, but they will affect the dynamics of the system; the NanC proteins will need to negotiate the complex free energy landscape created by these protein-lipid-protein interactions if they are to reach an energetically stable state through oligomerization.

This result may also be compared with studies of membrane protein interactions using more approximate (and hence more general) models and DPD simulations [36]. For example, such studies have suggested that changes in lipids may result in the modulation of mismatch-driven interactions of membrane proteins [45].

We identified a correlation between of the depth of the well in the free energy of association with the buried surface area at the interface of the two proteins. More generally, it has been suggested that oligomer stability of 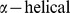 membrane proteins such as glycophorin A [22] and bacteriorhodopsin [46] may be correlated with the buried surface area at the interface. However, studies of the dimeric outer membrane protein OMPLA [15] failed to reveal such a correlation. This may reflect the role of lipids in OMPLA dimerization, confirming the need for detailed energy landscape calculations such as those presented herein.

Features of the methodology used in this work mean that care should be taken when interpreting the results. The treatment of solvents in the coarse-grained model is only approximate, so entropic contributions to solvation and lipidation/delipidation may not be captured as reliably as with a fully atomistic model. The nature of the coarse-grained model also does not enable us to separate out the contributions to the PMF due to energy and entropy, as is sometimes done using atomistic calculations. However, the observations we make here relating to the behaviour of lipids is mostly phenomenological and any quantitative observations are limited to relative comparisons between simulations of the same system. Furthermore, in choosing to look at a highly restrained system where the relative positions and orientations are restrained, we are also looking at the change in free energy along a narrow slice through configuration space. Although this path may be tightly defined, it is only by using such a highly restrained system that we are able to identify some previously unobserved behaviour, specifically the effect of protein-lipid-protein interactions on the free energy of protein dimerization. Such effects would usually be lost when averaging over a larger range of configurations.

The results presented here highlight some of the effects that contribute to the free energy of association for a bacterial outer membrane protein that undergoes non-specific oligomerization in a POPE bilayer. These processes will play a role in many protein-protein interactions, even those with some specific oligomerization modes, although in the latter case the non-specific interactions will likely be masked at close range by the specific interactions. We would expect the protein-lipid-protein interactions to be present in many membrane protein systems, as they seem to be determined by the underlying lipid-protein interactions. The PMF for the association of two OmpF trimers calculated by Casuso et al. [6] had a potential well that was approximately twice as deep as the ones we present here for NanC. However, the OmpF protein is much larger than NanC and the oligomerized proteins would therefore have a correspondingly larger buried surface compared to our NanC system.

Given the conclusions of this study, it will be of great interest to apply similar methods to those presented to calculate orientationally-dependent PMFs for a variety of other membrane proteins. Information obtained from PMFs, such as the orientational dependence of the free energy of association, are necessary for parameterizing yet coarser (i.e. more approximate) models (for example those of Yiannourakou et al. [33]), in order to enable simulation studies of the emergent properties of large, crowded and complex membrane models [47].

## Methods

### Using umbrella sampling to calculate PMFs

Umbrella sampling was used to obtain the PMF for each of the four orientational configurations while varying the inter-protein separation [32]. The umbrella sampling was performed using simulation windows in which one protein was restrained at relative positions with the desired inter-protein separation. We chose this measure as our reaction coordinate because it is a natural choice for characterizing the separation of two proteins and it would also enable the PMFs to be used to parameterize larger scale models, as done by Yiannourakou et al. [33].

To calculate the PMF from these individual biased simulation windows we employed the weighed histogram analysis method (WHAM) [48]. For the WHAM method to produce a converged PMF, we need to ensure that all points along the reaction coordinate are sufficiently sampled. This means that the histograms from the sampling of the reaction coordinate in each simulation window need to overlap with adjacent simulation windows and that these histograms are smooth. The effect of enforcing these requirements is that all points along the reaction coordinate are thoroughly sampled in multiple simulation windows. It is these considerations that determined our positioning of the umbrella sampling windows and the strength of the biasing potentials used in each.

For each orientational configuration, umbrella sampling windows were distributed at positions with varying inter-protein separation along a line connecting the centres of mass of the two proteins. The umbrella potential was applied to the centre of mass of each protein's 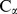 particles, restraining them at relative positions with the desired centre of mass separation. The sampling windows were distributed at 0.1 nm intervals from an inter-protein separation of 2.8 nm to 8 nm. In each of these simulation windows we applied a harmonic umbrella potential with a force constant of 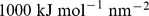. To improve the overlap of the histograms from adjacent simulation windows in the region of the local minima, which improved the resolution, additional simulation windows were used. These additional simulation windows were distributed at 0.05 nm intervals from a inter-protein separation of 2.8 nm to 4.5 nm, where the proteins were in close proximity. These closely separated windows had a stronger harmonic force constant of 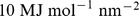, in order that we could better resolve the barriers surrounding the restrained local minima.

The lipidation/delipidation of the protein-protein complex at close range is a slow process. It is important that we adequately sample both the lipidated and delipidated state. To do so, we also performed simulations in which that interface was manually delipidated, with the intervening lipids returned to the bulk of the bilayer, and the system re-equilibrated. Manually delipidated simulations were carried out for the orientational configurations 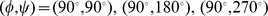, but were not required for 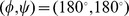 as no persistent lipidated state was observed. Delipidated simulation windows were distributed from an inter-protein separation of 2.8 nm to 3.7 nm for 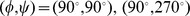, and to 4.0 nm for 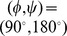, separated by 0.5 nm in all cases and using the stronger position restraint of 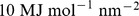.

### Orientational restraints

To analyse the free energy of association for specific relative orientations of NanC we had to ensure that we restrained their orientations in each simulation window, as well as their relative positions. This was achieved by applying a rotational potential to the 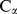 particles of each protein. The rotational potential for each protein acted around a vector in the 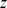 direction (approximately perpendicular to the plane of the membrane) through the protein's centre of mass. By applying a suitable rotational potential to the proteins, we were able to restrain their rotation without influencing the positional umbrella potential. Kutzner et al. [49] showed that we can virtually eliminate both the radial forces and forces parallel to the axis of rotation by using a restraining potential of the form
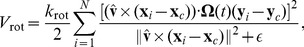
(1)where 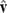 is a unit vector parallel to the rotation axis; 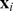 and 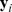 are the current and reference positions of the 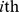 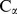 particle, respectively; 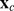 and 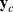 are the current and reference positions of the centre of mass of the 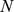 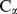 particles in each protein, respectively; 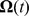 is a rotation matrix, which describes the motion of the potential; 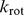 is the force constant for the rotational potential, and 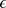 is a small constant required to avoid a singularity at the axis of rotation.

The application of this potential to the 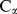 particles of each protein results in a purely rotational force (a torque) about the proteins' centres of mass that acts in an approximately perpendicular direction to the plane of the membrane. This rotational potential is implemented by the *enforced rotation* feature of the GROMACS MD simulation package, which at each time-step applies an appropriate translational force to each of the restrained particles in order to create the desired torque [49], [50]. The proteins were inserted into the membrane in the desired relative orientations for each configuration and so we wished to restrain their orientations, which was achieved by setting the rotation matrix, 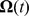, equal to the identity matrix. The reference positions for the 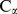 particles were taken from the initial protein structures at maximum separation. The force constant 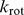 was set to 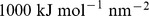, which kept rotational drift below 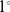, and the constant 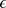 was set to 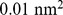. Kutzner et al. showed that values for 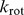 greater than 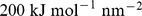 were shown to give good results for rotating the 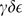 subdomain of 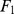 ATPase using the same value of 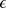 that we have used here [49].

### Molecular dynamics simulations

To calculate the PMF of association for two NanC proteins we employed coarse-grained molecular dynamics simulations of a bilayer system. The system consisted of the two coarse-grained proteins embedded in a symmetric bilayer formed from 424 coarse-grained POPE lipid molecules. The bilayer was solvated and counter ions were added to neutralize the system. The coarse-grained force field we implemented was a modified version of the MARTINI forcefield [29], [41], in which approximately four heavy atoms were mapped to each coarse-grained particle. This mapping can be seen between Figure 1A and Figure 1B. All simulations were run using GROMACS v4.6 ScalaLife 2012 (available from http://www.scalalife.eu) [50]. The simulations were performed under conditions of constant temperature (310 K) and pressure (1 bar) using a timestep of 40 fs. We have provided a GROMACS simulation configuration (*mdp*) file in the Supporting Information (Data S1) for a simulation window in which the protein restrained at relative positions corresponding to a centre of mass separation of 4 nm with a force constant of 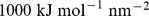.

Each of the simulation windows were equilibrated for between 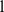 and 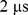. The production simulations consisted of at least 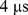 of simulation for each of the 0.1 nm separated simulation windows, where the applied umbrella potential force constant was 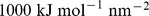. The length of simulation was increased if the PMF had not converged sufficiently. The convergence of each PMF was evaluated by comparing the PMFs obtained using non-intersecting subsets of production simulation data (see Figure S3). For each of the 0.05 nm separated simulation windows, where the larger force constant of 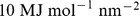 was applied, 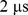 of production simulations were performed. We also performed 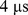 of production simulation for the manually delipidated simulation windows, which were separated by 0.05 nm. To combine the simulation data to obtain the PMFs we used the g_wham program, distributed with GROMACS [51], using a tolerance of 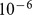.

### Predicting restrained metastable state locations from the lipid distribution around a single NanC

In an attempt to predict the location of the restrained metastable states we performed a 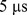 simulation of a single NanC protein freely diffusing in a POPE bilayer. This extended simulation consisted of a single coarse-grained NanC protein model (the same model used for the PMF calculations) embedded in a 25 nm square membrane constructed from coarse-grained POPE molecules. To analyse the lipid distribution around the single protein, we rotated each frame of the trajectory so that the NanC protein was aligned with its position at the start of the simulation. From this aligned trajectory we were able to calculate the position of the lipid particles in relation to the protein for the entire simulation. The particle density was calculated for a 6 nm square region around the NanC protein for each of the particles in the coarse-grained lipid molecules.

To calculate the protein density in a given direction, we calculated a linear projection of this two-dimensional density. For the case of the protein orientation configuration 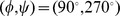, the direction we are interested in is the same for both of the proteins, as they have the same face oriented toward the other protein. This direction is marked by the dashed lines in Figure 3D. In order to characterize the lipid particle density in this direction, we projected the two-dimensional density onto a series of 4 nm lines emanating from the protein's centre of mass, at regular angular intervals, within the region marked by the dashed lines. The dashed lines represent an angular window of 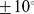 and the individual projection lines were separated by 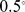.

To predict the location of the minimum and the local minima of Figure 2B, assuming the lipid behaviour corresponds to that shown in Figure 3D, we aligned the peaks of the mean lipid species plot (where the mean was taken across the linear projections for all coarse-grained lipid particles in both leaflets and is shown in Figure 3E) with those of the same plot overlaid with the x-axis reversed. For the case of a single intervening lipid we aligned the first peak with the first peak of the reversed plot (see Figure 3F). For the case of two intervening lipids we aligned the first peak with the second peak of the overlaid plot (see Figure 3G). Finally for the case of three intervening lipids, we aligned the first peak with the overlaid third peak and the second peak with the overlaid second peak (see Figure 3H).

### Calculating the buried surface area

To obtain the buried surface area of the proteins at various positions along the reaction coordinate, we analysed the surface area of the simulation windows with the higher translational restraining potential, 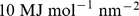, to enable the analysis of the surface area on a finer scale, using window separations of 0.05 nm instead of 0.1 nm. Using the higher force constant also ensured that the surface area was measured for a conformation that was sampled closer to the centre of the window; with the weaker force constant we would be measuring the surface area for conformations with separations that could differ significantly from the position of the window centre. All of the surface area calculations were carried out using the g_sas tool in GROMACS using 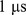 of production simulation trajectory.

## Supporting Information

Data S1**GROMACS configuration file.** A typical configuration file (.*mdp* file) for one of the umbrella simulation windows where the proteins are restrained at relative positions corresponding to a centre of mass separation of 4 nm with a force constant of 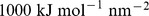.(TXT)

Figure S1 **View of the extracellular leaflet.** This is taken from the snapshot of the simulation shown in Figure 3A. Here we can see that the intervening region between the two proteins only contains the single lipid from the periplasmic leaflet, again shown in purple.
(TIFF)

Figure S2**A PMF calculated for two NanC proteins in POPE bilayer, which have not been orientationally restrained.** There are no observed local minima in this PMF. We calculated this PMF using the same simulation parameters as the orientationally-restrained PMFs, except the strength of the rotational potential, 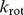, was set to zero. Each of the simulations windows were equilibrated for 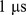 and then 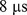 production simulations were used to calculate this PMF. The PMF possesses many of the same features as the rotationally restrained versions, such as a small energy barrier around 5–6 nm and a deep potential well, but it is otherwise smoothly varying.(TIFF)

Figure S3**Rotationally restrained PMFs calculated using non-intersecting subsets of the simulation data, which are used to asses the convergence of the PMFs in ****Figure 2****.** The four orientational combinations are for proteins angles of: 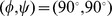; 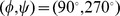; 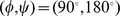; and 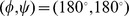 (in *A*, *B*, *C*, and *D*, respectively). To evaluate the convergence of the rotationally-restrained PMFs presented in this paper we calculated two PMFs for each orientational configuration, each using half of the simulation data. The data used for each PMF constituted non-intersecting subsets of the total simulation data using to calculate the PMFs presented in Figure 2. From these PMFs we can see that the two versions agree to a high degree. The depths of the two PMFs calculated for each orientational combination are approximately the same and the local minima are observed at the same locations. There are a few regions where they do not overlap precisely, but these discrepancies do not affect the conclusions of the paper.(TIFF)
