# Supplementary figures and images for: The Free Energy Landscape of Dimerization of a Membrane Protein, NanC
Source: PLoS Comput Biol. 2014 Jan 9;10(1):e1003417. doi: 10.1371/journal.pcbi.1003417 (PMC3886892; doi:10.1371/journal.pcbi.1003417)

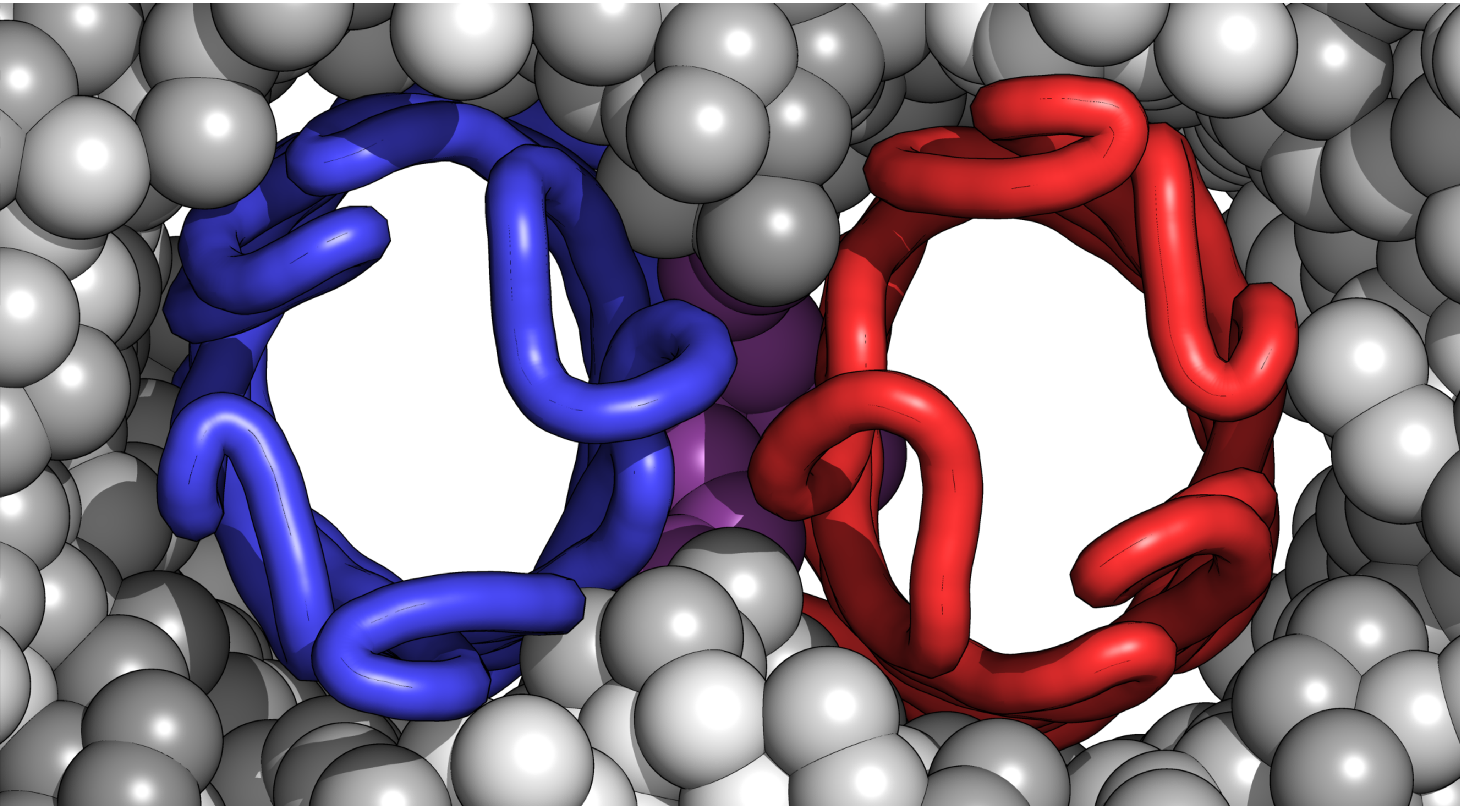

Supplement: Figure S1 — View of the extracellular leaflet. This is taken from the snapshot of the simulation shown in Figure 3A. Here we can see that the intervening region between the two proteins only contains the single lipid from the periplasmic leaflet, again shown in purple. (TIFF) [file pcbi.1003417.s002.tiff]

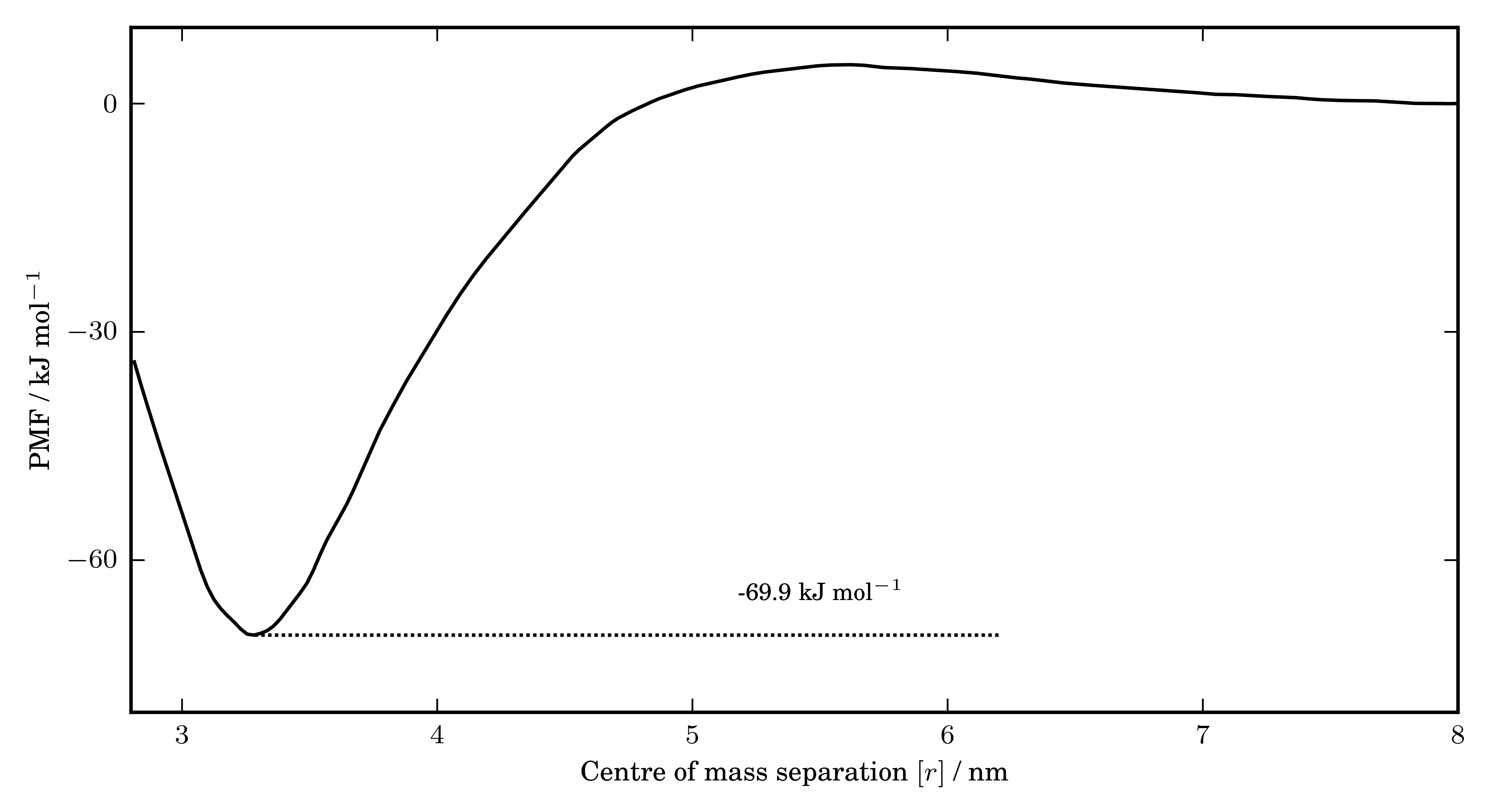

Supplement: Figure S2 — A PMF calculated for two NanC proteins in POPE bilayer, which have not been orientationally restrained. There are no observed local minima in this PMF. We calculated this PMF using the same simulation parameters as the orientationally-restrained PMFs, except the strength of the rotational potential, , was set to zero. Each of the simulations windows were equilibrated for and then production simulations were used to calculate this PMF. The PMF possesses many of the same features as the rotationally restrained versions, such as a small energy barrier around 5–6 nm and a deep potential well, but it is otherwise smoothly varying. (TIFF) [file pcbi.1003417.s003.tiff]

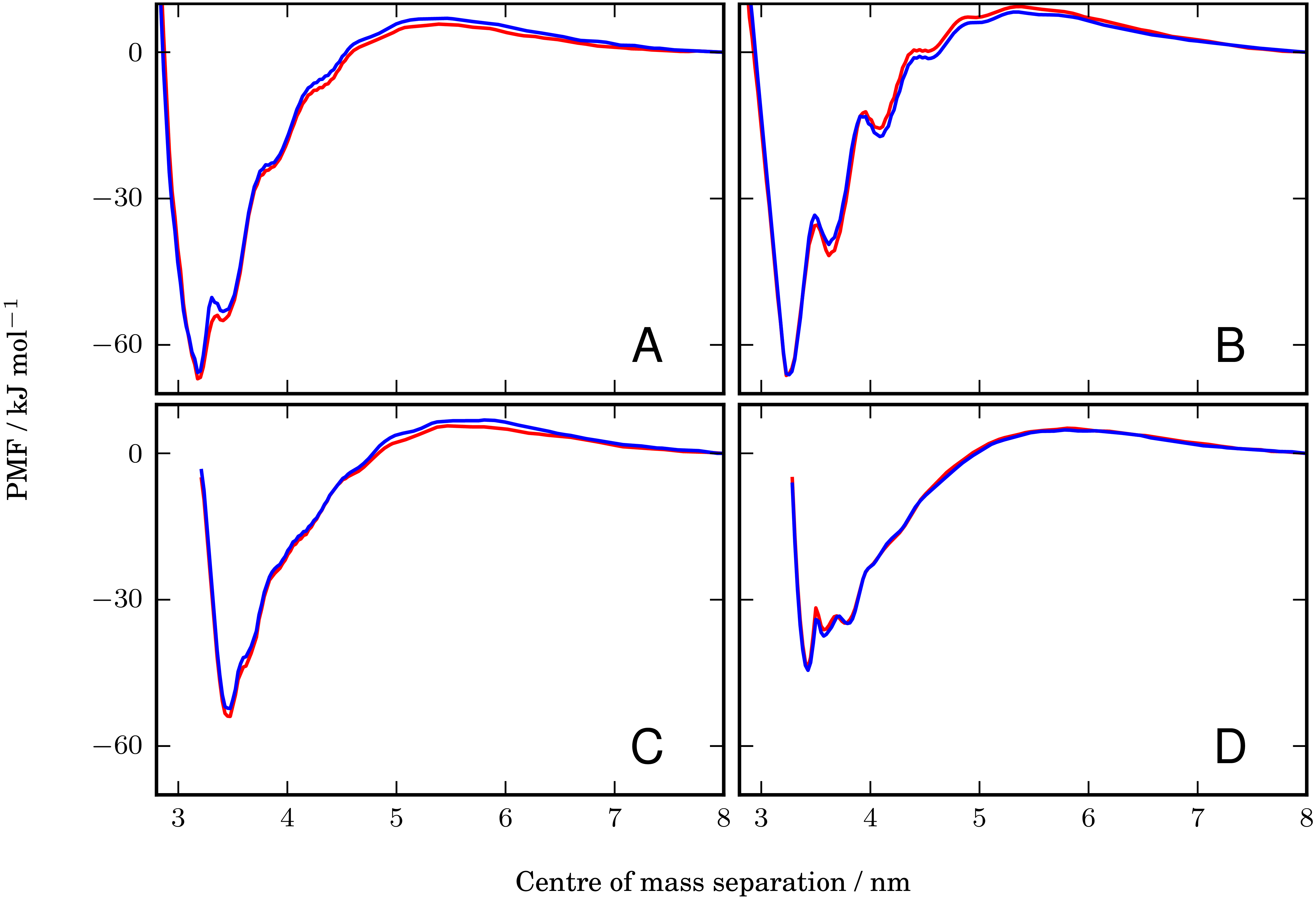

Supplement: Figure S3 — Rotationally restrained PMFs calculated using non-intersecting subsets of the simulation data, which are used to asses the convergence of the PMFs in Figure 2. The four orientational combinations are for proteins angles of: ; ; ; and (in A, B, C, and D, respectively). To evaluate the convergence of the rotationally-restrained PMFs presented in this paper we calculated two PMFs for each orientational configuration, each using half of the simulation data. The data used for each PMF constituted non-intersecting subsets of the total simulation data using to calculate the PMFs presented in Figure 2. From these PMFs we can see that the two versions agree to a high degree. The depths of the two PMFs calculated for each orientational combination are approximately the same and the local minima are observed at the same locations. There are a few regions where they do not overlap precisely, but these discrepancies do not affect the conclusions of the paper. (TIFF) [file pcbi.1003417.s004.tiff]
